# Carnosol controls the human glioblastoma stemness features through the epithelial-mesenchymal transition modulation and the induction of cancer stem cell apoptosis

**DOI:** 10.1038/s41598-017-15360-2

**Published:** 2017-11-09

**Authors:** Chiara Giacomelli, Simona Daniele, Letizia Natali, Caterina Iofrida, Guido Flamini, Alessandra Braca, M. Letizia Trincavelli, Claudia Martini

**Affiliations:** 1 Department of Pharmacy, University of Pisa, Via Bonanno 6, 56126 Pisa, Italy; 2 Centro Interdipartimentale di Ricerca “Nutraceutica e Alimentazione per la Salute”, University of Pisa, via del Borghetto 80, 56124 Pisa, Italy

## Abstract

A high cell proliferation rate, invasiveness and resistance to chemotherapy are the main features of glioblastoma (GBM). GBM aggressiveness has been widely associated both with a minor population of cells presenting stem-like properties (cancer stem-like cells, CSCs) and with the ability of tumor cells to acquire a mesenchymal phenotype (epithelial-mesenchymal transition, EMT). Carnosol (CAR), a natural inhibitor of MDM2/p53 complex, has been attracted attention for its anti-cancer effects on several tumor types, including GBM. Herein, the effects of CAR on U87MG-derived CSC viability and stemness features were evaluated. CAR decreased the rate of CSC formation and promoted the CSC apoptotic cell death through p53 functional reactivation. Moreover, CAR was able to control the TNF-α/TGF-β-induced EMT, counteracting the effects of the cytokine on EMT master regulator genes (Slug, Snail, Twist and ZEB1) and modulating the activation of miR-200c, a key player in the EMT process. Finally, CAR was able to increase the temozolomide (TMZ) anti-proliferative effects. These findings demonstrate that CAR affected the different intracellular mechanism of the complex machinery that regulates GBM stemness. For the first time, the diterpene was highlighted as a promising lead for the development of agents able to decrease the stemness features, thus controlling GBM aggressiveness.

## Introduction

Glioblastoma multiforme (GBM) is the most aggressive form of glioma (WHO grade IV) and displays strong infiltrating properties^1^. The first therapeutic choice is surgery, followed by the treatment with the alkylating agent, Temozolomide (TMZ). Nevertheless, the median survival of patients with GBM is only 2 years after diagnosis, due to the resistance to therapy and/or tumor recurrence^2,3^. The aggressive phenotype^4^, the invasiveness and the resistance to chemotherapy and radiotherapy^5,6^ of GBM have been correlated with the expression of stem cell markers^7,8^ and with the acquisition of a mesenchymal phenotype^9–11^. The tumor bulk contributing to the stemness of GBM includes cancer stem cells (CSCs) and cells with a mesenchymal phenotype, which are derived from the de-differentiation of cells with an epithelial phenotype. In this light, great interest in the discovery of novel therapeutic approaches that are able to target cancer cells with a stem phenotype has arisen.

The epithelial-mesenchymal transition, commonly known as the EMT, is an evolutionary process in which cells lose their epithelial features and acquire a mesenchymal phenotype through concerted and tightly regulated epigenetic and biochemical processes^12,13^. The EMT is indispensable in physiological processes such as wound healing and embryonic development. Conversely, in the cancer bulk, the induction of the EMT has been linked to the acquisition of a more stem-like phenotype^14^, which confers resistance to therapy, aggressive traits and an invasive phenotype to cells. The EMT have been widely implicated in the aggressiveness of different solid tumors^15^, including GBM^16–19^, and has been linked to frequent tumor recurrence and metastasis.

The GBM malignancy is also increased by the presence of a sub-population of cancer cells with extremely high tumorigenic potential: the CSCs. In the last decade, these cells have been isolated from a variety of cancers^20–23^, including GBM^24–28^. CSCs present properties of self-renewal, multipotent differentiation and the capacity to generate new tumors with the same heterogeneity as the original tumors. These cells contribute to the aggressiveness, frequent relapse and higher resistance to chemotherapy and radiotherapy of GBM^8^.

Several studies have identified correlations between the EMT and CSCs. Generally, CSCs are proposed to originate either from adult stem cells that have undergone a malignant change, or from differentiated cells (progenitor cells) that have acquired the ability to self-renew and de-differentiate into cancer cells with more stem-like properties^29–31^. Cancer cells that underwent the EMT exhibit a CSC-like phenotype, acquiring a greater stemness profile^32–34^. Although the exact link between the CSC-EMT and tumor progression is not clear, the discovery of novel agents that are able to eradicate these subpopulations of cells with stem-like properties has arisen as an important challenge in the development of effective GBM treatments. In the last years, several strategies have been pursued to target CSCs, such as induction of apoptosis, inhibition of self-renewal and chemoresistance-related pathways, or induction of their differentiation^35^.

In this scenario, phytochemicals have been shown to be promising as anti-cancer treatments, contributing to both the modulation of the EMT and the reduction of CSC viability^36–41^. Among the various phytochemicals with anticancer properties, the diterpene carnosol (CAR) has shown to have significant cytotoxic effects on several human cancer cell lines and animal models^42,43^. CAR is a naturally occurring phenolic diterpene found in several Mediterranean herbs and is a major component of rosemary (*Rosmarinus officinalis* L.)^42,43^. In a our recent study, CAR exerted an anti-proliferative effect on GBM through the inhibition of the MDM2/p53 complex and the functional reactivation of the p53 pathway^44^. Vergara *et al*. reported the ability of the diterpene to inhibit the EMT in ovarian cancer^43^. However, to the best of our knowledge, no data have been reported on the effects of CAR on CSCs and the EMT in glioma.

Herein, for the first time, the ability of CAR to modulate the EMT and affect the CSCs viability were evaluated in human GBM cell model. CAR decreased the expression of transcription factors implicated in the induction of the EMT, thus preventing the transition. Furthermore, CAR controlled the EMT transition affecting the expression of the intracellular small non-coding RNA miR-200c, which is a key regulator of the EMT and promotes the expression of stemness-related genes in CSCs^45–47^. Moreover, CAR promoted the CSC death increasing the effect of TMZ. The diterpene was also able to control the self-renewal of the CSCs by inhibiting the expression of stemness-related genes (*nanog, SOX2* and *Oct4*) CAR could represent a tool to better understand the mechanism that confers the highly aggressiveness to the brain tumors. Furthermore, the diterpene could represent the starting point for the development of more effective chemotherapeutic agents able not only to control the proliferation of the differentiated cells but also to affect the CSCs pool increasing their sensitivity to TMZ treatment.

## Results

### Experimental plan

As a representative GBM cell line, we used U87MG cells, which is an appropriate model to study the effects of the MDM2-p53 complex inhibitor CAR. In fact, the U87MG cells maintain a wild type status of p53, and are deficient for the tumour suppressor phosphatase and tensin homologue (PTEN) that leads to MDM2 nuclear accumulation, thus inhibiting p53 functions^48^.

CAR treatment decreases adherent U87MG cell proliferation with an IC_50_ values of 28.9 µM and 14.9 µM after 24 h or 48 h, respectively, due to the p53 reactivation^44^. P53 could affect other features of cancer cells, thus, in order to assess the CAR effects in adherent U87MG, a concentration lower than the anti-proliferative IC_50_ values was used.

Then, the CAR cellular activity and molecular mechanisms were explored in different human GBM derived stem cells (CSCs) expressing wild-type p53 (U87MG and U343MG) or mutated p53 (T98G)^48^.

### Effects of CAR on EMT process in GBM cells

#### Induction of the EMT in U87MG cells through inflammatory priming

**Figure 1:**
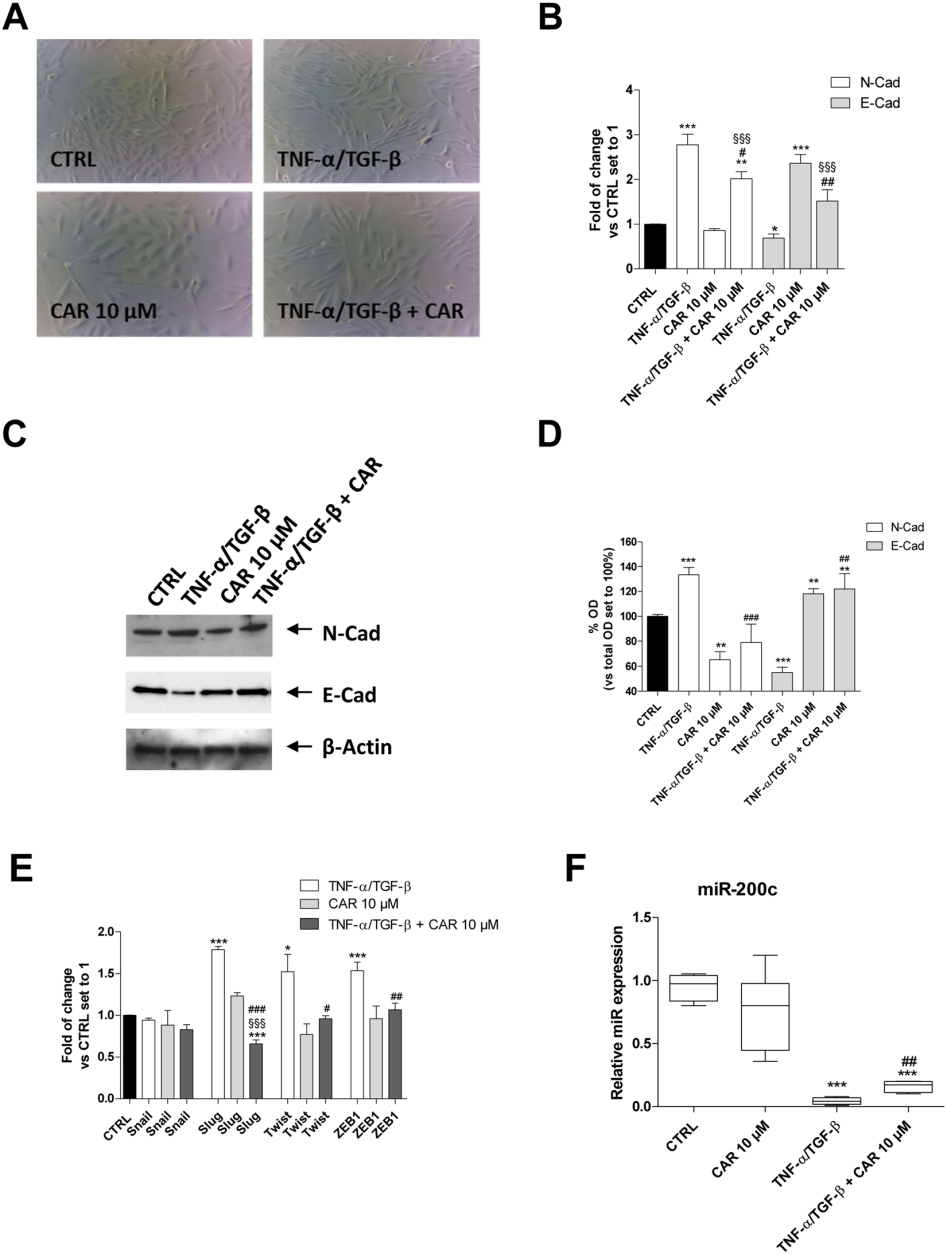
CAR modulation on the TNF-α/TGF-β1-induced EMT. U87MG cells were treated with TNF-α (10 ng/ml)/TGF-β1 (10 ng/ml) in the absence or the presence of CAR (10 µM) in complete medium for 48 h. (**A**) At the end of the incubation, representative images were taken. (**B**) Real Time RT-PCR analysis of the EMT markers. The data were the mean values from three different experiments. (**C**,**D**) U87MG cells were treated as described above, and the levels of the EMT markers were evaluated by Western blotting with the use of specific antibodies. One representative Western blot is presented (**C**). The bar graph (**D**) shows the densitometric analysis of the Western blot, which was performed using the ImageJ program. The data are presented as the means of three different experiments. (**E**) Real Time RT-PCR analysis of the transcription factors that act as master regulators of the EMT (Snail, Slug, Twist and ZEB1) (**F**) U87MG cells were treated as indicated, and the levels of miR-200c were quantified at the end of the incubation. The data are presented as the means of three different experiments each performed in duplicate. The significance of the differences was determined by one-way ANOVA, followed by Bonferroni’s post hoc test: *P ≤ 0.05, **P ≤ 0.01, ***P ≤ 0.001 vs. the control; ^#^P ≤ 0.05, ^##^P ≤ 0.01, ^###^P ≤ 0.001 vs. TNF-α/TGF-β1 alone; ^§§^P ≤ 0.01, ^§§§^P ≤ 0.001 vs. CAR alone.

Cytokines released in the tumor microenvironment are known to affect the phenotype of the cancer cells, leading to the acquisition of a higher stem grade^49^. Moreover, they could affect the cancer stem cell pool, prompting the maintenance of greater stem-like features. Typically, the EMT process is initiated by the action of different cytokines and extracellular stimuli^50^. Therefore, U87MG cells were induced to undergo the EMT by treatment with a mixture of the cytokines TNF-α (10 ng/ml) and TGF-β1 (10 ng/ml) for 48 h. First, a morphological analysis of the cells was performed (Fig. 1A). The TNF-α/TGF-β1 treatment induced a change in cell morphology: U87MG cells, which normally display an oval shape, showed an elongated shape with a fibroblast-like appearance.

The morphological changes, which are characteristic of cells undergoing the EMT, are accompanied by a shift in the expression from epithelial genes to a mesenchymal gene repertoire^19,50^. Accordingly, challenging cells with the TNF-α/TGF-β1 mixture modified the expression of EMT markers, leading to a significant increase in the expression of the mesenchymal marker and a concomitant decrease in the expression of the epithelial marker E-cadherin (2.77 ± 0.69- and 0.69 ± 0.08-fold vs. CTRL, respectively; Fig. 1B). The data were confirmed at the protein level by Western blots for N- and E-cadherin (Fig. 1C,D).

The EMT is regulated by the activity of four main master regulator transcription factors (TFs): Snail, Slug, Twist and ZEB1^19,50^. Herein, the TNF-α/TGF-β1 treatment did not significantly affect the expression of the Snail gene. Conversely, the cytokine mixture significantly increased the expression of the other TFs, as compared to the control cells (Fig. 1E). Taken together, the data highlighted the ability of the inflammatory microenvironment to induce the EMT in U87MG cells.

#### CAR modulates the induced EMT in U87MG cells

In the literature, there are no reports on the effect of the rosemary component on the EMT. Thus, the effects of CAR in U87MG human glioblastoma cell induced-EMT were investigated. The cells treated with CAR (10 µM) exhibited a change in the expression of surface EMT markers. In particular, treatment with the diterpene alone significantly increased both the expression of the E-cadherin both at mRNA and protein level, with a simultaneous slight decrease in N-cadherin expression (Fig. 1B–D). CAR interfered with the EMT, even if the effect was not sufficient to modulate the profile of the EMT master regulators that are tightly controlled by several feed-back mechanisms. Furthermore, CAR induced a significant change in the expression of epithelial and mesenchymal markers, at the mRNA (N-cad 2.0 ± 0.1, P ≤ 0.05 and E-cad 1.5 ± 0.3 P ≤ 0.01 vs. TNF-α/TGF-β1 treatment) and protein levels (N-cad 68.4 ± 12.4, P ≤ 0.001 and E-cad 73.5 ± 7.3 P ≤ 0.05 vs. TNF-α/TGF-β1 treatment). These data agreed with the morphological changes observed in the cells after the CAR treatment (Fig. 1A). Thus, CAR negatively regulated the TNF-α/TGF-β1-induced EMT, resulting in the maintenance of a reduced mesenchymal phenotype of the human U87MG cells.

#### CAR modulated the genes regulating the cytokine-induced EMT in adherent U87MG cells

CAR alone did not significantly modulate the expression of the TFs Snail, Slug, Twist and ZEB1, which have been identified as master regulators of the EMT. However, CAR counteracted the up-regulation of Slug, Twist and ZEB1 induced by the cytokine mixture (Fig. 1E).

Among the different regulatory pathways involved in the EMT, the expression of specific miRNAs has attracted substantial interest. miR-200c is a key negative regulator of the EMT^51^. Thus, the intracellular expression of miR-200c was evaluated in response to TNF-α/TGF-β1 stimuli in the absence or presence of CAR (Fig. 1F). As expected, the TNF-α/TGF-β1 mixture induced a significant decrease in miR-200c expression (0.04 ± 0.01-fold vs. CTRL, P ≤ 0.001). CAR significantly counteracted the effect of the cytokine mix (0.16 ± 0.02-fold vs. TNF-α/TGF-β1), even if it was not able to completely restore the control profile. The analysis of the correlation with the expression of the EMT markers indicated that the epigenetic control of miR-200c could be one of the mechanisms underlying the inhibitory effects of CAR on GBM cell mesenchymal transformation. Notably, CAR did not affect miRNA expression in the absence of the cytokine mixture.

### Effects of CAR on CSC biological properties

#### The stem-like features of U87MG cells are influenced by the CAR treatment

**Figure 2:**
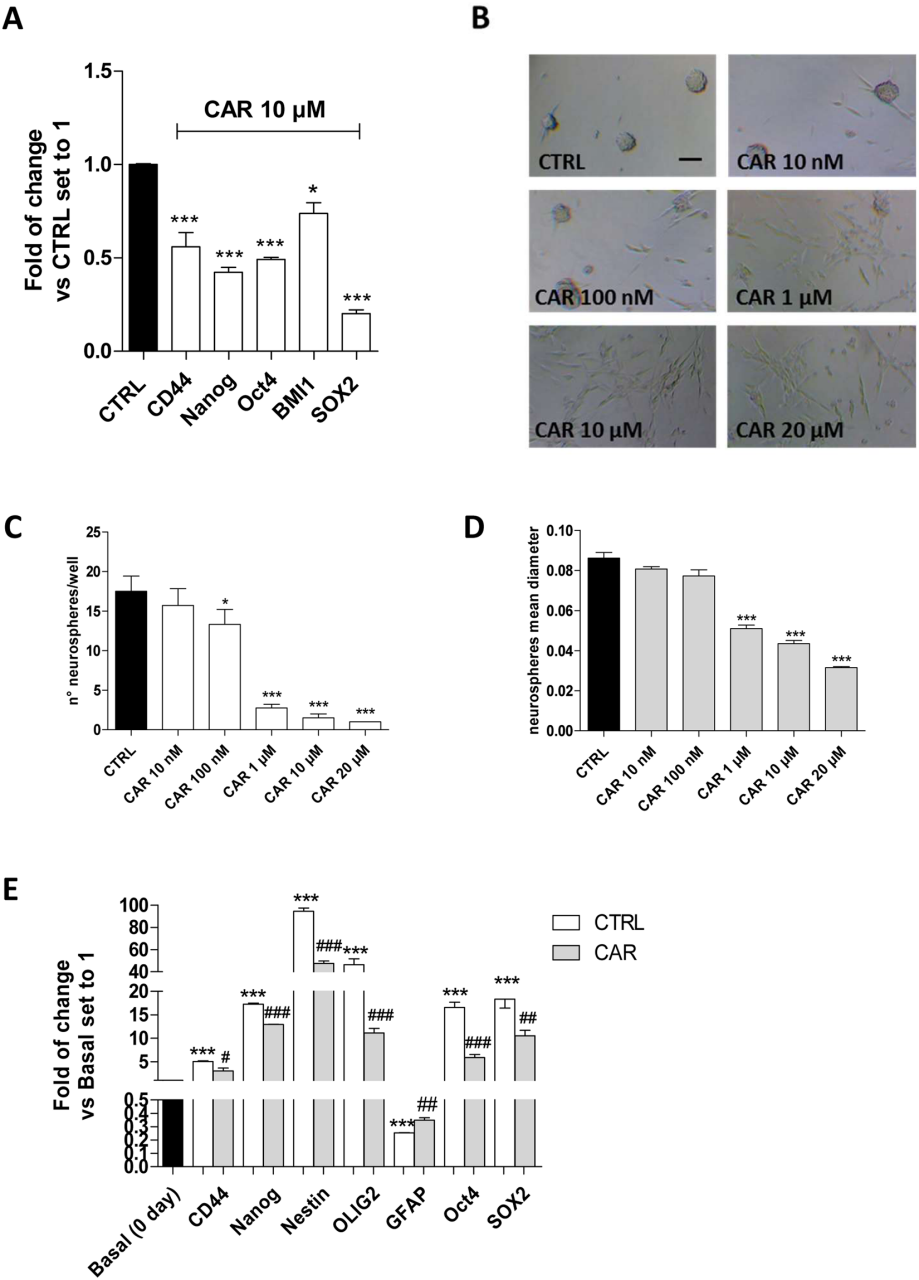
Effects of CAR on stemness gene expression and CSC sphere formation. (**A**) The U87MG cells were treated with DMSO (CTRL) or CAR (10 µM) in complete medium for 48 h. At the end of the incubation, a Real Time RT-PCR analysis of stem gene expression was performed. The data are presented as the mean values from three different experiments each performed in duplicate. (**B**–**E**) U87MG cells were incubated with DMSO or CAR (10 nM–20 µM) in a defined serum-free NSC medium for 9 days. (**B**) Representative image. The number of the newly formed spheres (**C**) and the mean diameter (**D**) were scored using the ImageJ program. The data represent the mean values from three pictures from two independent experiments. (**E**) Cells were treated as described above and a Real Time RT-PCR analysis of the stem cell markers or differentiated cell markers was performed. The data are presented as the fold of change vs. the expression at basal (day 0) level, before the treatment with the NSC medium, and are the mean values from three different experiments. The significance of the differences was determined by one-way ANOVA, followed by Bonferroni’s post hoc test: *P ≤ 0.05, **P ≤ 0.01, ***P ≤ 0.001 vs. the CTRL; ^#^P ≤ 0.05, ^##^P ≤ 0.01, ^###^P ≤ 0.001 vs. 0 day.

U87MG cells, as well as other GBM cells, are heterogeneous and normally include a small fraction of CSCs^52^. The expression of stem cell markers was evaluated to assess the effect of CAR on the stem-like nature of the differentiated cancer cell line (Fig. 2A). CAR significantly decreased the expression of the stemness genes CD44, Nanog, Oct4, BMI1 and SOX2, when it was used at a 10 µM concentration, demonstrating that the diterpene affected the stemness of the CSC fraction that is normally present in culture lines.

Then, the ability of CAR to affect the process by which glioblastoma CSCs are formed was examined. For this purpose, adherent U87MG cells were switched to a serum-free NSC medium and the cells were allowed to growth for additional 9 days in the presence or absence of different concentration of CAR (10 nM–20 µM) (Fig. 2B–D). CAR was able to reduce the number of spheres in a dose-dependent manner, indicating its capacity to alter the process by which stem cells are generated within tumor bulk (Fig. 2C). Furthermore, CAR decreased the diameter of the newly formed spheres suggesting the compound’s ability to inhibit the proliferation of glioblastoma stem cells (Fig. 2D). The CAR concentration used here was ten times lower than the IC_50_ value reported for the anti-proliferative activity^44^ to discriminate the apoptotic effect on U87MG cells and the real inhibitory effect on CSC formation.

In order to confirm the CAR inhibitory effects on CSC formation, the expression of stem cell (CD44, Nanog, Nestin and OLIG2) and differentiated cell (GFAP) marker genes were evaluated. CAR significantly decreased the mRNA expression of stem cell markers and increased the expression of the GFAP mRNA (Fig. 2E), thus demonstrating its ability to modulate the de-differentiation of GBM cancer cells.

#### CAR decreased CSC viability and promoted TMZ-induced anti-proliferative effects

**Figure 3:**
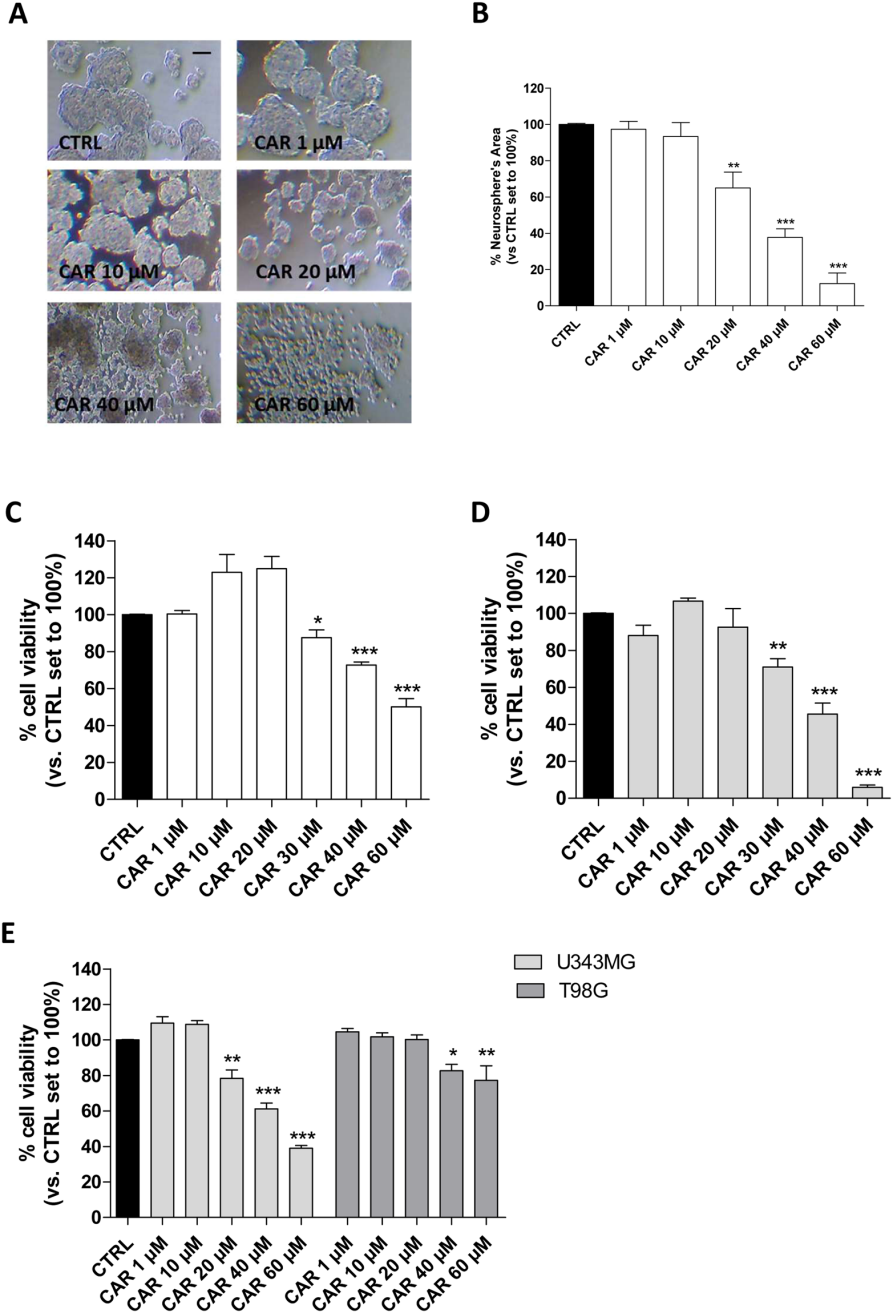
Effects of CAR on the morphology and viability of the CSC. U87MG-derived, U343MG-derived and T98G-derived CSCs were treated with complete NSC medium containing DMSO (CTRL) or the indicated concentrations of CAR (100 nM-60 µM) for 3 or 7 days. (**A**) Representative images were captured after 7 days of incubation, and the area of the culture plates occupied by the spheres (**B**) was scored using the ImageJ program. The data are presented as the mean values from three independent experiments. For each experimental condition, five pictures were analyzed. U87MG-derived CSCs were treated as described above for 3 days (**C**) or 7 days (**D**). Then, cell proliferation was measured using the MTS assay. (**E**) U343MG-derived and T98G-derived CSCs were treated as described above for 7 days. Then, cell proliferation was measured using the MTS assay. The data are presented as the mean values from three independent experiments, each performed in duplicate. The significance of the differences was determined by one-way ANOVA, followed by Bonferroni’s post hoc test: *P ≤ 0.05; **P ≤ 0.01, ***P ≤ 0.001 vs. the CTRL.

Based on the role of the CSC subpopulation in GBM invasiveness and recurrence, we examined the effects of CAR on pre-formed glioblastoma derived-CSCs. The formation of neurospheres in U87MG, U343MG and T98G cell cultures *in vitro* was induced by a specific neural stem-cell (NSC) medium^53,54^. Consistent with literature data^53–56^, the spheres obtained using U87MG, U343MG and T98G (Fig. S1, Figs 2 and 3) included significantly higher levels of the stem cell markers CD133, Nanog, Nestin, OLIG2, CD44, SOX2, Oct4, BMI1 and STAT3 a smaller percentage of GFAP compared with the adherent counterpart (Figs S1, 2 and 3). These data were confirmed by the decrease of GFAP protein expression levels and the increase of Nestin expression, a differentiation and stem markers, respectively (Fig. S1). Moreover, CSCs presented a greater ability to form spheres with respect to adherent cells (54.6% CSC, 10.8% U87MG, P ≤ 0.001; 39.4% CSC, 9.6% U343MG, P ≤ 0.001; 38.6% CSC, 8.3% T98G, P ≤ 0.001), indicating that CSCs retain a clonogenic potential. Finally, different CSCs were confirmed to exhibit a significant higher resistance to TMZ with respect to adherent counterpart (Figs S1E, 2E and 3E). Collectively, these data support the reliability of CSC isolation, as we previously reported^54^.

The effects of CAR on CSC morphology were evaluated by quantifying both the mean area occupied by the cells in culture plates and the possible outgrowth of cellular processes. CAR significantly reduced the area occupied by the floating spheres (Fig. 3A,B), without producing outgrowth of cellular processes.

Then, the proliferation rate of CSCs was analyzed. As depicted, CAR induced a time-dependent inhibition of U87MG-CSC proliferation (Fig. 3C,D). The effect appeared to be concentration-dependent, with an IC_50_ value of 37.5 ± 5.9 µM after 7 days of treatment (Fig. 3D, Fig. S4A) with a maximum effect of 92.7 ± 3.5%. Similarly, CAR was able to decrease the viability of the U343MG-CSCs with an IC_50_ value of 33.5 ± 2.6 µM after 7 days of treatment (Fig. 3E, SI Fig. S4B) but with a maximum effect of only 62.3 ± 1.4%. Conversely, CAR exerted only a slight effect on T98G cell viability with a maximum reduction of 22.7 ± 8.2% (Fig. 3E). The different response of the GBM-CSCs on CAR could be ascribed to the p53 status. CAR inhibition of MDM2/p53 complex confer to the diterpene a lower effect on T98G cell that possess a mutated protein, in accordance with the previously reported effect of CAR on respective differentiated cells^44^.

**Figure 4:**
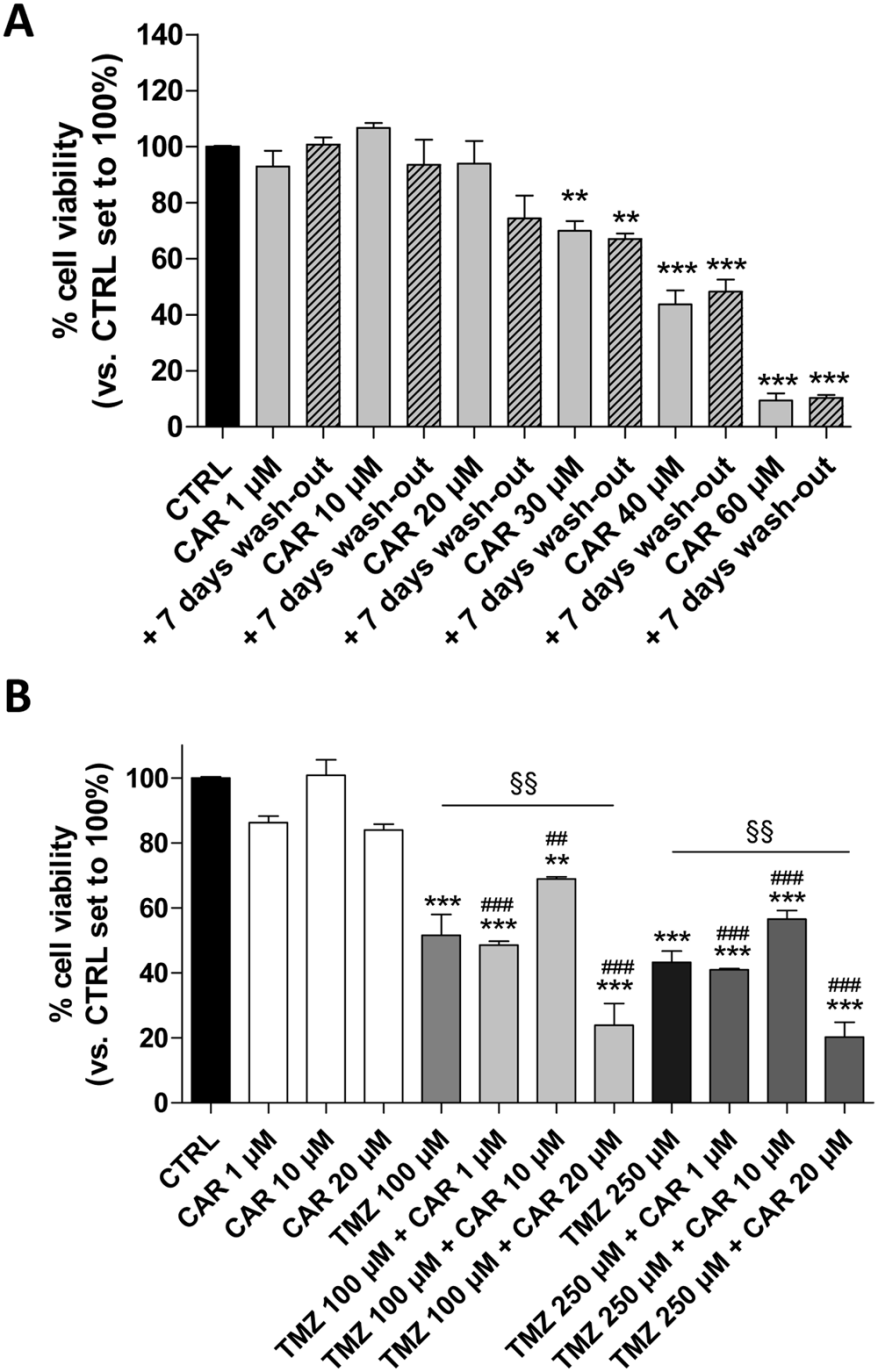
CAR sensitize CSCs to TMZ treatment. U87MG-derived CSCs were treated with complete NSC medium containing DMSO (CTRL) or the indicated concentrations of CAR (100 nM–60 µM) for 7 days. (**A**) The drug-containing media were replaced with fresh drug-free NSC media and the cells were cultured for an additional 7 days. Then, cell proliferation was measured using the MTS assay. (**B**) U87MG-derived CSCs were treated in NSC medium with CAR (1–20 µM) and/or TMZ (100–250 µM) for 7 days. Then, cell proliferation was measured using the MTS assay. The data are presented as the mean values from three independent experiments, each performed in duplicate. The significance of the differences was determined by one-way ANOVA, followed by Bonferroni’s post hoc test: *P ≤ 0.05; **P ≤ 0.01, ***P ≤ 0.001 vs. the CTRL.

Next, we assessed whether CAR-treated cells could resume growing after the drug was removed. CSCs were challenged with CAR (1–60 µM) for 7 days and then washed-out for additional 7 days in drug-free medium. The percentages of proliferating cells after the drug removal did not significantly increase, suggesting their overall inability to recover normal growth (Fig. 4A). Accordingly, CAR has also been reported to produce a long-lasting effect on U87MG cells^44^. Thus, the diterpene produced long-lasting and anti-proliferative effects on the U87MG-derived CSC subpopulation.

To determine if CAR could potentiate the anti-proliferative effect of the alkylating agent temozolomide (TMZ), in cancer stem cells, the diterpene and TMZ were combined together. Challenging U87MG-CSCs for 7 days with TMZ (100–250 µM) plus CAR at different concentrations produced a significant reduction of cell proliferation rate (Fig. 4B). These results suggested that CAR enhances TMZ anti-proliferative activity in CSCs, in accordance with its effects on adherent U87MG^44^.

#### P53 functional reactivation of CAR induced the cell cycle blockade and activated the apoptotic process

**Figure 5:**
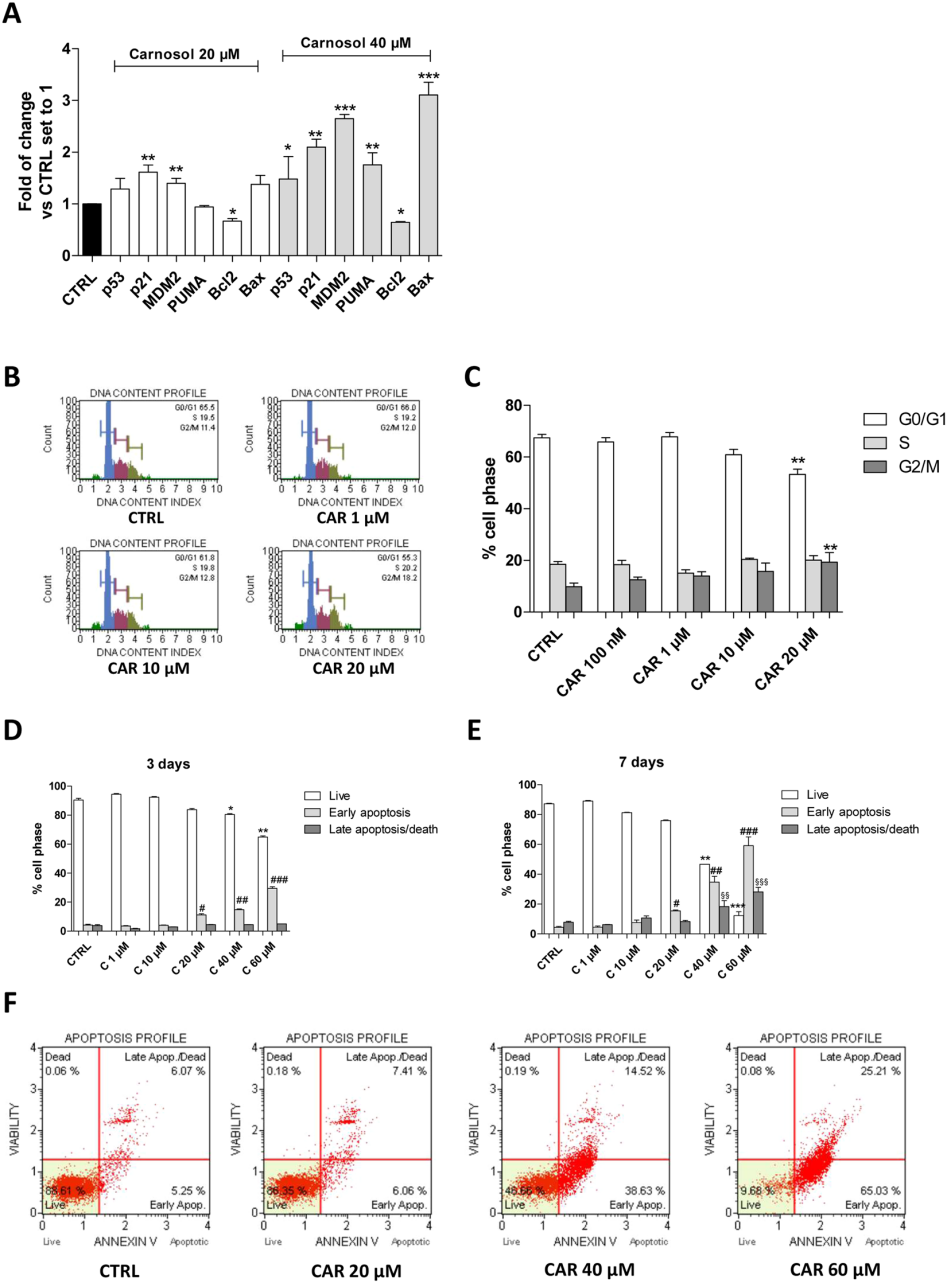
CAR effects on the cell cycle progression and apoptosis induction of U87MG-CSCs. U87MG-derived CSCs were treated with DMSO (CTRL) or CAR for 3 or 7 days. (**A**,**B**) At the end of the treatments, the cell cycle was analyzed. The subpopulations of cells in the different phases of the cell cycle are shown. The data are presented as the mean values from three different experiments. (**C**–**E**) Cells were collected and the amount of phosphatidylserine externalization was evaluated using the Annexin V staining protocol. The distribution of the live, early and late apoptotic cells. (**E**) Representative plots are presented. The data are presented as the mean values from three different experiments. The significance of the differences was determined by one-way ANOVA, followed by Bonferroni’s post hoc test: *P ≤ 0.05, **P ≤ 0.01, ***P ≤ 0.001 vs. the CTRL; ^#^P ≤ 0.05, ^##^P ≤ 0.01, ^###^P ≤ 0.001 vs. CTRL early apoptotic cells.

Next, the mechanisms underlying the reduced CSC proliferation were evaluated. Functional reactivation of p53 has been widely correlated to increases in specific p53-related genes^57–60^. Thus, a real-time RT-PCR analysis of the p53-related genes was performed after the U87MG-derived CSC subpopulation were treated with CAR (20–40 µM) for 3 days. CAR was able to significantly increase the mRNA levels of all of the p53 target genes (Fig. 5A): p21 (with a maximum increaseof ≈2.1-fold), PUMA (with a maximum increase of ≈1.7-fold), and MDM2 (with a maximum increase of ≈2.6-fold). The expression of the pro-apoptotic ‘multi-domain’ Bcl-2 family member Bax and the anti-apoptotic protein Bcl-2 was assessed. As expected, CAR treatment caused a significant increase in Bax (maximum increase of ≈3.1-fold) and a concomitant decrease in Bcl-2 (maximum decrease of ≈0.5-fold) (Fig. 5A). The results are in accordance with data showing CAR modulation of p53 levels and the functional reactivation of p53-mediated gene transcription in GBM and other cell lines^44,61–63^.

Challenging CSCs with 10 µM CAR for 7 days did not affect the cell cycle. In contrast, when a higher concentration of CAR was added (20 µM), a decrease in the DNA content in G1/G0 phases and a concomitant increase in the number of cells in G2/M phases was observed. Therefore, CAR blocked cell cycle in G2 phase (Fig. 5A,B), consistent with previously reported data from U87MG cells^44^.

The inhibition of MDM2/p53 lead to the functional reactivation of p53 that switch on the apoptotic machinery^44^. Thus, the CAR apoptotic activity was evaluated (Fig. 5C–E). CAR induced clear, significant signs of U87MG-CSC apoptosis after 3 days of treatment, which became more evident after 7 days of incubation (Fig. 5C–E). Surprisingly, the 3-day treatment with CAR was already sufficient to induce the significant phosphatidylserine externalization in the absence of 7-AAD staining, thus denoting the signs of the early phase of apoptosis (Fig. 5C).

#### Self-renewal of CSCs is reduced by CAR

**Figure 6:**
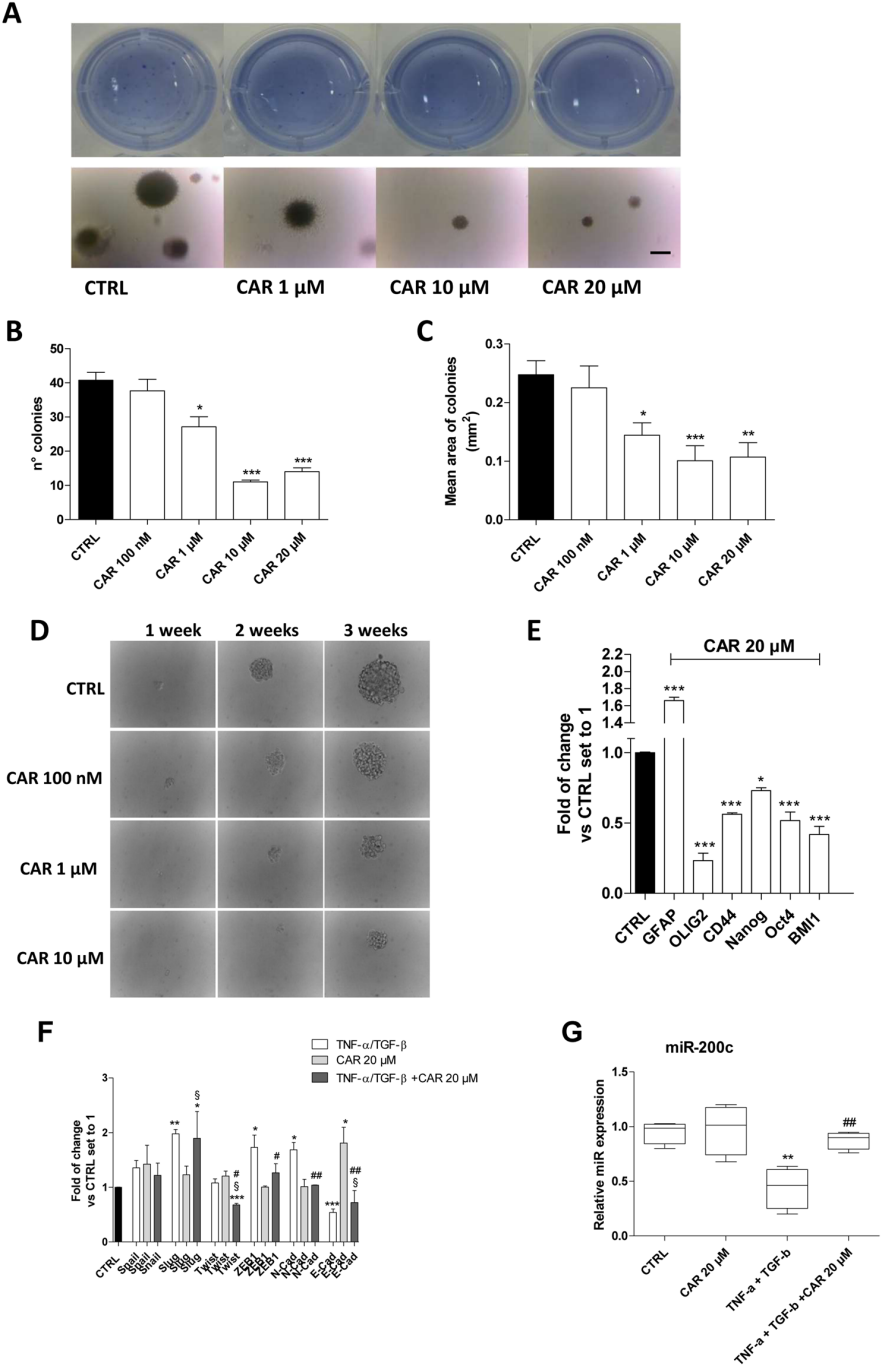
Effects of CAR on the frequency of U87MG sphere-forming cells and self-renewal capacity. (**A**–**C**) U87MG-derived CSCs were grown in 0.36% agar in NSC medium in the presence of DMSO (0,5%, CTRL) or CAR (1–20 µM) for 14 days. At the end of the incubation, representative microscopic images (**A**) were captured at ×4 magnification before and after crystal violet staining. The number (**B**) and mean area (**C**) of the colonies were scored using the ImageJ program. Only colonies with diameters greater than 60 µm were counted. The data are presented as the mean values from three independent experiments. For each experimental condition, five pictures were analyzed. (**D**) A single U87MG-derived CSC was cultured in a 96-well plate and maintained in NSC medium for 3 weeks in the absence (CTRL) or presence of different concentrations of CAR (100 nM–10 µM). Images were captured at different time points. (**E**) The U87MG-derived CSCs were treated with DMSO (CTRL) or CAR (20) in NSC medium for 7 days. At the end of the incubation, a Real Time RT-PCR analysis of the expression of different genes was performed. (**F**) U87MG-CSCs were treated with TNF-α (10 ng/ml)/TGF-β1 (10 ng/ml) in the absence or the presence of CAR (20 µM) in NSC medium for 7 days, then a real Time RT-PCR analysis of the transcription factors (Snail, Slug, Twist and ZEB1) was performed. (**G**) U87MG cells were treated as indicated, and the levels of miR-200c were quantified at the end of the incubation. The data are presented as the means of three different experiments. The significance of the differences was determined by one-way ANOVA, followed by Bonferroni’s post hoc test: *P ≤ 0.05, **P ≤ 0.01, ***P ≤ 0.001 vs. the control; ^#^P ≤ 0.05, ^##^P ≤ 0.01, ^###^P ≤ 0.001 vs. TNF-α/TGF-β1 alone; ^§§^P ≤ 0.01, ^§§§^P ≤ 0.001 vs. CAR alone.

The main feature of CSCs is their self-renewal capacity^9^. Herein, the effects of CAR on the formation of CSC colonies were evaluated using the soft-agar colony-forming assay (Fig. 6A–C). CAR inhibited the colony-forming capacity of CSCs in a dose-dependent manner. CAR significantly inhibited the number of newly formed colonies (sphere numbers 14.0 ± 1.1 CAR 20 µM, 40.7 ± 2.4 CTRL, P ≤ 0.001; Fig. 6B), highlighting its ability to block the self-renewal of CSCs. Furthermore, CAR decreased the mean diameter of the colony in a dose-dependent manner (0.247 ± 0.03 mm^2^ CTRL, 0.107 ± 0.02 mm^2^ CAR 20 µM, P ≤ 0.01; Fig. 6C), thus decreasing the self-renewal rate of CSCs, as well.

A tumor sphere formation assay was performed to deeply assess the ability of CAR to decrease the amount of stem cell in neurospheres and limit their self-renewal. CAR, in a dose dependent manner, inhibited the ability of CSCs to form spheres (Fig. 6D), as revealed by the decrease in the colony diameter. Furthermore, the CAR treatment significantly decreased the efficacy of sphere formation (88.5% CTRL, 33.3% CAR 10 µM, P ≤ 0.001).

Thus, CAR decreased the total number of stem cells and slowed down the self-renewal capacity of the CSCs.

#### CAR affects stemness signature of U87MG-derived CSCs

Self-renewal capacity is tightly regulated by several stem markers^64,65^. Because CAR modulated the self-renewal properties of CSCs, the effects on stemness gene expression were evaluated (Fig. 6E). CAR increased the expression of the differentiation marker GFAP (P ≤ 0.001) and decreased the expression of stem cell markers (CD44, Nanog, Oct4, BMI1 and OLIG2). Thus, CAR not only affected the viability of CSCs but also could modulate their stemness gene signatures.

Nevertheless, the CSCs in glioblastoma have a greater mesenchymal profile rather than an epithelial one. After induction, this phenotype can be shifted toward an even more mesenchymal phenotype in through an EMT-like process. Therefore, CSCs were treated with the TNF-α/TGF-β1 mixture, and the expression of EMT markers and the transcription factors acting as master regulators was evaluated (Fig. 6F). The mixture caused a significant increase in N-cadherin expression (1.68 ± 0.13-fold vs. CTRL, P ≤ 0.05) concomitant with a decrease in E-cadherin expression (0.54 ± 0.06-fold vs. CTRL, P ≤ 0.001). The effect on the TFs implicated in the EMT was less than the effect observed in U87MG cells. Indeed, only Slug expression was significantly increased after treatment with the cytokine mixture compared to the control (1.98 ± 0.08-fold vs. CTRL, P ≤ 0.01). CSCs exposed to TNF-α/TGF-β1 only partially underwent the EMT, probably due to the already high expression of stemness factors.

CAR did not significantly affect the expression of EMT markers and transcription factors in CSCs in the absence of the cytokine mixture. Conversely, CAR (20 µM, a concentration lower the IC_50_ values on U87MG-CSC proliferation) counteracted the EMT elicited by TNF-α/TGF-β1 by reducing the expression of mesenchymal markers (Fig. 6F).

In parallel, the intracellular expression of miR-200c, a key negative regulator of the EMT, was also evaluated in CSCs. The TNF-α/TGF-β1 mixture significantly decreased miRNA expression (P ≤ 0.01), consistent with the data obtained in U87MG cells (Fig. 1F). CAR (20 µM) counteracted the effects of TNF-α/TGF-β1 on miR-200c expression (P ≤ 0.01); on the contrary, CAR alone produced only a slight increase in the intracellular miRNA levels in the absence of the cytokines.

Thus, CAR was able to interfere with the TNF-α/TGF-β1-induced EMT also in CSC population, despite a higher concentration was needed, in accordance with the less sensitiveness of CSCs to chemotherapeutic agents.

## Discussion

**Figure 7:**
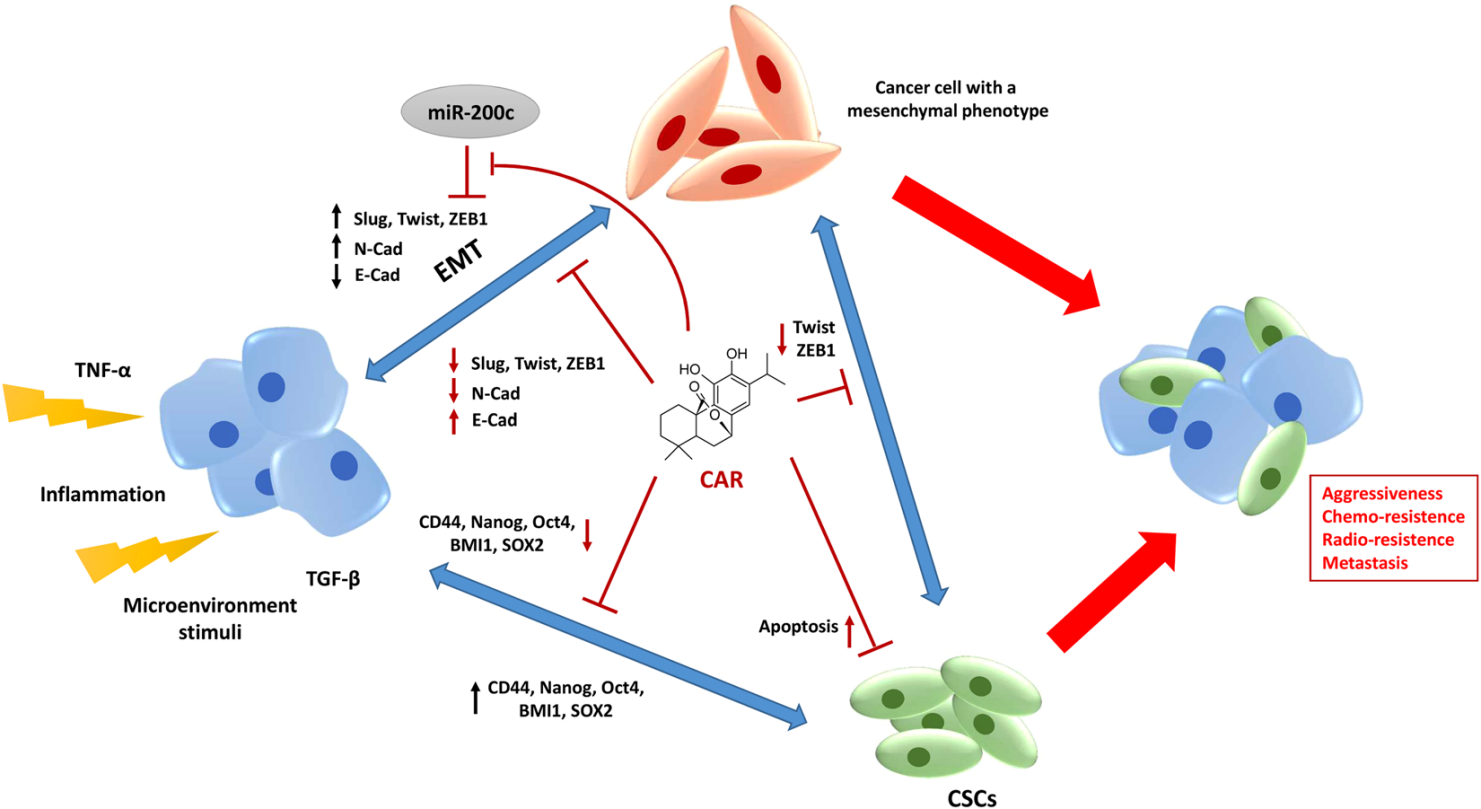
Schematic overview of the EMT and CSC connection in the tumor progression and the role of CAR in the control of glioblastoma stemness. The tumor microenvironment and extracellular stimuli play crucial roles in CSCs formation and EMT transformation. Both these processes are at the basis of the increase of glioblastoma stemness and favoring the increase of cancer aggressiveness and recurrence. CAR controls the GBM stemness interacting with multiple intracellular pathways decreasing the EMT transition, the CSC formation and the CSC maintenance.

The natural diterpene CAR, a MDM2/p53 complex inhibitor, decreased the stemness phenotype of human U87MG cells by interfering with both the EMT and the stem-like CSC biology. Specifically, CAR affected the EMT by decreasing the expression of stemness regulator genes in both U87MG cells and their derived stem-like CSCs. In addition, CAR induced CSC apoptosis through the functional reactivation of p53, leading to the reduction of proliferation and irreversible stem cell death, at least at high concentrations (Fig. 7).

The cancer bulk was principally divided into two cell types consisting of the differentiated cancer cells and the undifferentiated CSCs. The glioblastoma-derived CSCs possess self-renewal capacity and are able to differentiate into a specular cancer bulk. These cells may be responsible for relapse and metastasis by giving rise to new cancer. The acquisition of the mesenchymal phenotype and the presence of CSCs have been widely correlated with an increase in cancer aggressiveness in GBM; nevertheless, the link between the EMT, the acquisition of the mesenchymal phenotype and the detection of CSCs in the GBM bulk is still unclear^9,25–28^.

Recently, our group has reported the anti-proliferative activity of CAR in human GBM cell lines^44^. CAR inhibits the MDM2/p53 complex producing a p53 functional reactivation in adherent U87MG cells^44^. As reported in several recent papers, the oncosuppressor protein, p53, participates in decreasing cancer stemness features, regulating the CSCs fate^66,67^, and suppressing the EMT^68^. Based on the regulatory effect of CAR on p53, the influence of the diterpene on the EMT, the stemness features of U87MG cells, and CSC viability was evaluated in this study.

The tumor inflammatory microenvironment plays a crucial role in facilitating cancer aggressiveness and may induce the EMT. As reported by several previous studies, at the molecular level, the EMT is influenced by several cytokines, chemokines and growth factors within the inflammatory microenvironment that trigger the expression of different stemness genes, favoring the transition to a mesenchymal phenotype^13–15,50,69–71^. Accordingly, based on accumulating evidence, treatments with different factors that induce the EMT lead to an enrichment of cells with cancer stem-like features^32–34^. Although the exact mechanism of the EMT is still unclear, all these aspects are related to the aggressiveness of GBM and the escape from a cure.

Herein, a TNF-α/TGF-β mixture was used to induce the peculiar trait of the EMT in U87MG cells. The treatment of U87MG cells with the cytokine mixture increased the levels of several transcription factors identified as master regulators of the EMT, including Slug (also known as Snail2), Twist and zinc-finger E-box-binding (ZEB1). In parallel, as shown in other solid tumors^72–74^, the cytokine mixture reduced the expression of the epithelial marker E-cadherin and increased the expression of the mesenchymal marker N-cadherin, consistent with the induction of the mesenchymal phenotype.

In adherent U87MG cells, CAR significantly counteracted the TNF-α/TGF-β1 EMT induction. These effects may reflect the decrease in U87MG cell migration and the functional reactivation of p53 after the cells were exposed to CAR. Indeed, the ability of p53 to reduce metastasis has been widely linked to the negative regulation of fundamental factors involved in the initiation and maintenance of the EMT^45,75^. Notably, CAR was not able to perturb the EMT machinery per se, as revealed by the expression of cell surface markers or the expression of transcription factors involved in the EMT. These data are consistent with the effects of CAR on the MDA-231 breast cancer cell line^43^, in which the diterpene did not influence the cell phenotype. Conversely, in the same study, the compound reversed the EMT induced by cytokines. In this scenario, CAR not only interferes with CSC features by impairing the stem cell phenotype but also decreases the induction of the EMT.

Based on several lines of evidence, different miRNAs, particularly the miR-200 family, play a crucial role in regulating the EMT^45,76^. Among the miR-200 family, the main miRNA involved in cancer progression and the EMT is miR-200c. ZEB1, which directly represses the epithelial phenotype, is a well-known and prominent gene target of miR-200c^77,78^. Furthermore, ZEB1 itself negatively regulates miRNA expression in a feed-back loop. More interestingly, p53 negatively regulates miR-200c expression. Based on this evidence, we investigated the effects of CAR on miR-200c expression. CAR did not directly affect miR-200c or ZEB1 expression, but rather counteracted the TNF-α/TGF-β1-induced regulation of miR-200c in U87MG cells. Thus, CAR contributes to block the switch to a mesenchymal phenotype induced by the inflammatory microenvironment by reducing the aggressive cancer phenotype.The acquisition of stem-like properties is linked to the activation of several genes, such as CD44, BMI1, Nanog, Oct4, and SOX2, which are expressed in embryonic stem cells, cancer cells, and cancer stem cells. These genes are dysregulated in several cancers, and their modulation may be the basis for new, innovative anti-cancer therapies mainly directed toward the cancer stem cell bulk^79^. CD44 has been widely used as a marker for CSCs and it has been implicated in the adhesion, motility, proliferation, and cell survival of several cancers. Indeed, CD44 and the B-cell-specific Moloney murine leukemia virus insertion site 1 (BMI1) support the stem cell state in both cancer cells and embryonic stem cells. Moreover, the suppression of CD44 expression has been reported to decrease the formation of tumors and spheres^80^. The homeobox-containing transcription factor Nanog, the POU domain-containing transcription factor Oct4 and the HMG domain-containing transcription factor SOX2 play a crucial role in CSC maintenance^81^. Herein, the ability of CAR to modulate the expression of these stemness genes was demonstrated in both differentiated U87MG cells and more markedly in U87MG-derived CSCs. The ability of CAR to interfere with Nanog, Oct4, SOX2, CD44 and BMI1 expression is consistent with the effects of other natural compounds (e.g., adriamycin and diflourinated curcumin) to control the cancer stem cell bulk and the aggressiveness of glioma and pancreatic adenocarcinoma^82,83^.

CAR induced a decrease in the expression of the CD44 gene. This effect could be likely related to the inhibitory effect of CAR on MDM2/p53 complex and the increase of intracellular p53 levels. Accordingly, it has been demonstrated that p53 regulate stemness by directly repressing CD44 expression^75^.

BMI1 is overexpressed in various cancers and regulates several intracellular pathways implicated in cell proliferation (p16/Rb and/or p14^ARF^/MDM2/p53 pathways), invasion (activation of the Akt/GSK3β/Snail pathway) and self-renewal (NF-kB-Nanog pathways)^84,85^. Consistent with the ability of CAR to decrease BMI1 expression, the compound also exhibited anti-proliferative effects and reduced invasiveness and self-renewal of the CSCs.

The modulation of EMT master regulators, the control of CSC stem genes and the modulation of miRNA expression are correlated with an increase of cancer chemo-sensibility, the reduction of CSC formation and a decrease in CSC self-renewal properties^86^. In previous reports, natural compounds with anti-cancer properties were shown to decrease the sphere-forming ability of GBM cell lines^87,88^. Accordingly, CAR interfered with CSC formation by decreasing the expression of stemness markers of glioma CSCs, such as OLIG2^89^. More interestingly, CAR decreased the viability of U87MG-derived CSCs by inducing apoptosis, even if it was not able to induce the evident differentiation of the stem cells. Finally, CAR also reduced the frequency of stem cells in the sphere and the self-renewal capacity of CSCs. These findings are consistent with the concomitant decrease in the expression of the stem cell genes (SOX2, Oct4 and Nanog). In fact, the expression of SOX2 and Oct4 has been directly linked to a higher frequency of sphere-initiating cells in different cancer cell lines^90,91^. Globally, these data highlighted the ability of CAR to interfere with the expression of stemness-related genes, leading to a decrease in stemness features accompanied by anti-proliferative effects on human GBM cells with stem-like properties. TMZ is an alkylating agent that is used as a first-line chemotherapeutic agent in glioblastoma. Accumulating *in vitro* and *in vivo* data have shown that different natural compounds enhance the efficacy of chemotherapy and radiotherapy in various cancers by regulating different intracellular pathways^92^. CAR is able to induce apoptosis in chemoresistant ovarian cancer cells^93^ and radiosensitises melanoma cells after radiotherapy^94^. The ability of CAR to sensitize GBM-CSCs cells to TMZ was demonstrated for the first time.

In the discovery of new chemotherapeutic drugs, the safety profile could represents a limitation. The induction of apoptosis and the cell cycle blockade could affect the selectivity of anti-tumor agents on cancer cells versus non-malignant cells. Notably, it has been reported that the CAR anti-proliferative effects is preferentially directed towards cancer cells, as reported in both animal and *in vitro* models^42,95^. Furthermore, CAR presented a favourable therapeutic window in glioblastoma cells as we previously reported^44^.

Thus, CAR not only affects the viability of differentiated GBM cells, as previously reported, but also interferes with the diverse processes implicated in cancer resistance and aggressiveness, such as CSC formation, proliferation and self-renewal. Interestingly, CAR decreased the influence of the cancer microenvironment by reducing the cytokine-induced EMT that underlies the acquisition of the mesenchymal phenotype. Nevertheless other *in vitro* and *in vivo* studies are fundamental to extend our results and clarify the CAR cellular mechanisms, the CAR ability to reactivate the p53 functionality promoting CSC proliferation control and decreasing EMT was highlighted for the first time. Thus, we can speculate that the discovery of a compound targeting different features of the cancer bulk, which increase its aggressive traits, may represent a promising starting point for the development of more effective drugs, particularly for the treatment of cancer with a high grade of complexity and heterogeneity, such as GBM.

## Materials and Methods

### Material

U87MG cells are a human glioblastoma cell line (WHO grade IV) and was purchased from the National Institute for Cancer Research of Genoa (Italy). The human T98G and U343MG cell lines (WHO grade IV) were obtained from the American Type Culture Collection (USA) and CellLines Service GmbH (Germany), respectively. Cell line was controlled for DNA profiling. All other reagents were purchased from commercial sources and were of the highest commercially available grade. CAR was purchased by Sigma Aldrich, (Cat. C9617).

### Cell lines and CSC isolation

U87MG and T98G cells were maintained in RPMI medium supplemented with 10% FBS, 2 mM L-glutamine, 100 U/mL Penicillin, 100 mg/mL Streptomycin and 1% non-essential amino acids (NEAA) at 37 °C in 5% CO_2_. U343MG cells were maintained in Minimum essential medium Eagle with 2 mM l-glutamine adjusted to contain 1.5 g/L sodium bicarbonate and supplemented with 10% FBS, 100 U/mL penicillin, 100 mg/mL streptomycin, 1% non-essential amino acids and 1.0 mM sodium pyruvate at 37 °C in 5% CO_2_.

The isolation of derived CSCs was performed as previously described^96^. Briefly, approximately 2.5 × 10^6^ cells were suspended in 1 mL of serum-free Neural Stem Cell (NSC) medium. After 3–5 days of culture, the CSCs (called “neurospheres”) were collected, suspended in NSC medium and plated for the assays. The derived-CSCs was characterized as previously reported^96,97^. For the long-term treatment of cells, NSC or complete medium containing drugs was replaced every two to three days.

### mRNA extraction and Real Time RT-PCR analysis

U87MG cells (3.5 × 10^3^ cell/cm^2^) and derived-CSC cells (40 spheres/500 µl) were treated with DMSO (CTRL) or CAR (at the reported concentrations) in the absence or the presence of Tumors necrosis factor α (TNF-α, 10 ng/ml) and Trasforming growth factor β1 (TGF-β, 10 ng/ml) for indicated times. Then, cells were collected, and total RNA was extracted using Rneasy® Mini Kit (Qiagen) according to manufacturer’s instructions. 500 ng of RNA were transformed in cDNA using i-Script cDNA synthesis kit (BioRad, Hercules, USA) following manufacturer’s instructions. Real-time RT-PCR reactions mix consisted of: 25 µL Fluocycle® II SYBR® (Euroclone), 1.5 µL of both 10 µM forward and reverse primers, 3 µL cDNA, and 19 µL of H_2_O. Reactions were performed for 40 cycles using this temperature profiles: 98 °C for 30 seconds (initial denaturation); T_ann_ (Table 1) for 30 seconds (annealing); and 72 °C for 3 seconds (extension). Primer used were reported in Table 1 ^98^. β-actin was used as the housekeeping gene. The mRNA levels for each sample were normalized against β-actin mRNA levels, and relative expression was calculated by using Ct value. The melting curve analysis and gel electrophoresis were performed to test the PCR specificity.

**Table 1:** Primers Used for Real-Time RT-PCR.

Gene	Primer nucleotide sequences	Product size (base pairs)	Annealing Temperature
CD44	FOR: 5′-CCGCTATGTCCAGAAAGGA-3′ REV: 5′-CTGTCTGTGCTGTCGGTGAT-3′	195 bp	55 °C
CD133	FOR: 5′-TCCACAGAAATTTACCTACATTGG -3′ REV: 5′CAGCAGTTCAAGACGCAGATGACCA-3′	251 bp	61 °C
Nanog	FOR: 5′-CATGAGTGTGGATCCAGCTTG-3′ REV: 5′-CCTGAATAAGCAGATCCATGG-3′	192 bp	55 °C
Oct4	FOR: 5′-CTCACCCTGGGGGTTCTATT-3′ REV: 5′-CTCCAGGTTGCCTCTCACTC-3′	230 bp	55 °C
BMI1	FOR: 5′-CCTGATGTGTGTGCTTTGTGG-3′ REV: 5′-TCATTAGAGCCATTGGCAGCA-3′	291 bp	55 °C
SOX2	FOR: 5′-CATGAAGGAGCACCCGGATT-3′ REV: 5′-ATGTGCGCGTAACTGTCCAT-3′	186 bp	55 °C
Nestin	FOR: 5′-CAGCGTTGGAACAGAGGTTGG-3′ REV: 5′-TGGCACAGGTGTCTCAAGGG-3′	282 bp	61 °C
OLIG2	FOR: 5′-CAGAAGCGCTGATGGTCATA-3′ REV: 5′-TCGGCAGTTTTGGGTTATTC-3′	208 bp	55 °C
GFAP	FOR: 5′-GCTCCGGAGACCCCTTCCA-3′ REV: 5′-GACAACCGCCACTCAACTAGC-3′	287 bp	52 °C
STAT3	FOR: 5′-GGCATTCGGGAAGTATTGTCG-3′ REV: 5′-GGTAGGCGCCTCAGTCGTATC-3′	318 bp	55 °C
N-Cad	FOR: 5′-AGGGGACCTTTTCCTCAAGA-3′ REV: 5′-CAATGTCAATGGGGTTCTCC-3′	246 bp	55 °C
E-Cad	FOR: 5′-AGGGGTTAAGCACAACAGCA-3′ REV: 5′-GGGGGCTTCATTCACATCCA-3′	395 bp	55 °C
Snail	FOR: 5′-AAGATGCACATCCGAAGCCA-3′ REV: 5′-CATTCGGGAGAAGGTCCGAG-3′	237 bp	55 °C
Slug	FOR: 5′-TGGTTGCTTCAAGGACACAT-3′ REV: 5′-GTTGCAGTGAGGGCAAGAA-3′	66 bp	55 °C
Twist	FOR: 5′-ACGAGCTGGACTCCAAGATG-3′ REV: 5′-CACGCCCTGTTTCTTTGAAT-3′	290 bp	55 °C
ZEB1	FOR: 5′-CCCTTGAAAGTGATCCAGCCA-3′ REV: 5′-AGACCCAGAGTGTGAGAAGCG-3′	354 bp	55 °C
MDM2	FOR: 5′-TCTAGGAGATTTGTTTGGCGT-3′ REV: 5′-TCACAGATGTACCTGAGTCC-3′	125 bp	55 °C
p21	FOR: 5′-TGCCGAAGTCAGTTCCTTG -3′ REV: 5′-CATGGGTTCTGACGGACATC-3′	134 bp	55 °C
BAX	FOR: 5′-TTTGCTTCAGGGTTTCATCC-3′ REV: 5′-CAGTTGAAGTTGCCGTCAGA-3′	245 bp	55 °C
PUMA	FOR: 5′-GAGGAGGAACAGTGGGC-3′ REV: 5′- CTAATTGGGCTCCATCTCGG-3′	198 bp	55 °C
p53	FOR: 5′-CTTTGAGGTGCGTGTTTGTG-3′ REV: 5′-GTGGTTTCTTCTTTGGCTGG-3′	161 bp	55 °C
Bcl-2	FOR: 5′-GAGGATTGTGGCCTTCTTTG-3′ REV: 5′-ACAGTTCCACAAAGGCATCC-3′	171 bp	55 °C
β-actin	FOR: 5′-GCACTCTTCCAGCCTTCCTTCC-3′ REV: 5′-GAGCCGCCGATCCACACG-3′	254 bp	55 °C

### Western blotting analysis of EMT markers expression

U87MG cells (3.5 × 10^3^ cell/cm^2^) were treated with DMSO (CTRL) or with CAR (10 µM) for 48 h, and then 200 μl RIPA buffer were added for 60 min at 4 °C to lyse the cells. 50 μg of total proteins was diluted in Laemmli solution, resolved by SDS-PAGE (7.5%), transferred to PVDF membranes and probed overnight at 4 °C with primary anti-E-cadherin antibody (diluted 1:200; sc-7870; Santa Cruz Biotechnology) or anti-N-cadherin antibody (diluted 1:200; sc-7939; Santa Cruz Biotechnology) or β-actin antibody (diluted 1:1000; MAB1501, Merck KGaA, Darmstadt, Germany). The primary antibody was detected using anti-rabbit IgG light chains conjugated to peroxidase (diluted 1:10000; 12–348; Millipore). The peroxidase was detected using a chemioluminescent substrate (ECL, Perkin Elmer), and the images were acquired by photographic film or by LAS4010 (GE Health Care Europe, Uppsala, Sweden). Immunoreactive bands were quantified performing a densitometric analysis with Image J Software.

### miRNA quantification analysis

miRNA was extracted from U87MG or derived-CSCs, treated as indicated, using the miRNeasy Mini Kit (Qiagen, Valencia, CA) following the manufacturer’s protocol. The expression of miRNAs was quantified using Taqman real-time reverse transcription RT–PCR assays following the manufacturer’s protocol (Applied Biosystems, Foster city, CA). In brief, 20 ng of total RNA was reverse transcribed and 6 ng of complementary DNA was used in each well for real-time RT–PCR. Each PCR reaction was performed in duplicate or triplicate. The quantification was performed using RNU6B Assay ID 001093 and hsa-miR-200c Assay ID 002300 (Applied Biosystems, Foster city, CA). The RNU6B was used as reference. The miRNA levels for each sample were normalized against RNU6B levels, and relative expression was calculated by using Ct value.

### Neurosphere formation assay

The ability of U87MG cells monolayer to initiate neurosphere formation was assessed as previously described^96^. Briefly, U87MG cells were seeded in 96 well plate at a density of 2 × 10^4^ cells/well in serum-free NSC medium and incubated with DMSO (0.5%, CTRL) or CAR (10 nM–20 μM) for 9 days without disturbing the plates and without replenishing the medium. At the end of the incubation time, images of the neurospheres were taken. Three wells were analysed and three images of each well were captured. The number and the mean diameter of the newly formed neurospheres were counted using the ImageJ program (version 1.41; Bethesda, MD, USA).

### CSC viability and quantification of neurosphere area

The U87MG-derived, U343MG-derived and T98G-derived CSCs were seeded (40 spheres/well) and treated with different CAR concentrations (1–40 µM) alone or in the presence of TMZ (100–250 µM) for 7 days. Then, cell proliferation was evaluated using the MTS assay (CellTiter 96® AQueous One Solution Cell Proliferation Assay kit; Promega) according to manufacturer’s instruction. The absorbance at 490 nm was measured with an automated plate reader (Victor Wallac 2, Perkin Elmer). For wash-out experiments, CSCs were treated with CAR (1–40 µM) for 7 days. Then, medium-containing drugs was replaced by fresh medium, and cells were allowed to growth for additional 7 days. Finally, MTS assay were performed to assess the cell proliferation in accordance to the manufacture instructions. Sigmoid dose-response curves were generated using GraphPad 5.0, from which the IC_50_ values were derived. Analysis of the neurosphere areas was performed as previously described^96^. Briefly, photographs of the neurospheres were taken at days 7. Three different wells were analysed for each condition, and 10 images of each well were captured. The area occupied by neurospheres that had formed was quantified using the ImageJ program.

### CSCs apoptosis and cell cycle analyses

For apoptosis measurement, CSCs were treated with DMSO (CTRL) or CAR for 7 days. Then, the percentages of living, apoptotic and dead cells were quantified and analysed by Muse™ Cell Analyzer (Merck KGaA, Darmstadt, Germany)^96^. The live, early apoptotic and late apoptotic/dead cells were discriminated using the staining with Annexin V and 7-Aminoactinomycin D (7-AAD).

For the cell cycle analysis, CSCs were treated with DMSO (CTRL) or CAR for 7 days. The quantification of the percentage of cells in the different cell phases was performed using the Muse™ Cell Analyzer^96^.

### Self-renewal assessment

Clonogenic and soft-agar colony forming assays were performed. For the clonal dilution assay, CSCs were dissociated and seeded at dilutions of 1 cell/well in NSC medium. Wells that contained a single cell were identified with microscopic observation, and the cells were maintained in NSC medium in the absences (CTRL) or presence of CAR (100 nM–10 µM). After 7, 14, or 21 days, colony formation was scored. The percentage of cells that formed spheres was determined by the following equation: (Y*(n)*/X*(n)*) *100 where X*(n)* is the number of wells in which a single cell was present and Y*(n)* is the number of wells in which one neurosphere developed from a single cell. The mean percentage of wells containing one neurosphere was measured and the mean diameter was evaluated using ImageJ program.

For the soft-agar colony formation assay, CSCs were dissociated, and 500 µl of 1 × 10^3^ cells in NSC medium containing 0.3% agar (low melting temperature agarose, Sigma-Aldrich) was plated on a layer of 500 µl of the same medium containing 0.6% agar in a 24 well plate. The plates were fed weekly with 0.2 ml of NSC medium. Two weeks after plating, the colonies were stained with 0.005% crystal violet, and photographs of the stained colonies were taken. The image were analysed using ImageJ program.

### Statistical analysis

The Graph-Pad Prism program (GraphPad Software Inc., San Diego, CA) was used for data analysis and graphic presentation. All data are the mean ± SEM of at least three different experiments. Statistical analysis was performed by one-way analysis of variance (ANOVA) with Bonferroni’s corrected t-test for post-hoc pair-wise comparisons. P < 0.05 was considered statistically significant.

## Electronic supplementary material

Supplementary Information
