# Supplementary material for: Carnosol controls the human glioblastoma stemness features through the epithelial-mesenchymal transition modulation and the induction of cancer stem cell apoptosis
Source: Sci Rep. 2017 Nov 9;7:15174. doi: 10.1038/s41598-017-15360-2 (PMC5680298; doi:10.1038/s41598-017-15360-2)
Supplement: Supplementary file 1 — Supplementary Information [file 41598_2017_15360_MOESM1_ESM.doc]

**Carnosol controls the human glioblastoma stemness features through the epithelial-mesenchymal transition modulation and the induction of cancer stem cell apoptosis.**

Chiara Giacomelli^1^, Simona Daniele^1^, Letizia Natali^1^, Caterina Iofrida^1^, Guido Flamini^1^, Alessandra Braca^1,2^, M. Letizia Trincavelli*^1,2^, Claudia Martini^1,2^

^1^ Department of Pharmacy, University of Pisa, Via Bonanno 6, 56126 Pisa, Italy;

^2^ Centro Interdipartimentale di Ricerca "Nutraceutica e Alimentazione per la Salute", University of Pisa, via del Borghetto 80, 56124 Pisa, Italy

***Corresponding author:**

M. L. T. Phone: +39-050-2219523; Fax: +39-050-2219609; e-mail: maria.trincavelli@unipi.it.

**Figure S1**

**Supplementary Figure 1**. Characterization of CSCs derived from adherent U87MG cells. (A) Representative bright field microscope images showing U87MG-CSCs, bar correspond to 100 µm. (B,C) Nestin and GFAP expression were evaluated by Western blotting with the use of specific antibodies. One representative Western blot is presented (B). The bar graph (C) shows the densitometric analysis of the Western blot, which was performed using the ImageJ program. (D,E) The total RNA was extracted from adherent U87MG cells and from the derived CSCs. The relative mRNA quantification of the stem cell markers (D) and of the astrocyte marker (E) was performed by Real-time RT-PCR. (F,G) CSC or U87MG adherent cells were seeded at different cell/well density, after 14 days the formed neurospheres were scored. (F) The graphs represent the logarithm of non-responding wells observed for the indicated cells/well density and (G) the total number of neurospheres formed for the indicated cells/well density (right panel). (H) U87MG adherent cells or U87MG-CSCs were dissociated single cells were grown in 0.36% agar in NSC medium for 14 days. At the end of the incubation, representative microscopic images were captured at x4 magnification before and after crystal violet staining. (I) U87MG cells or U87MG-CSCs were incubated with TMZ (250 µM) for 72 h. Then, cell proliferation was measured using the MTS assay. The data are presented as the mean values from three independent experiments, each performed in duplicate. ***P ≤ 0.001 vs U87MG. ### P ≤ 0.001 vs the relative effect in U87MG cells.

**Figure S2**

**Supplementary Figure 2**. Characterization of CSCs derived from U343MG cells. (A) Representative bright field microscope images showing U343MG-CSCs, bar correspond to 100 µm. (B) The total RNA was extracted from adherent U343MG cells and from the derived CSCs. The relative mRNA quantification of the stem cell markers (CD133, Nanog, Nestin and OLIG2) and of the astrocyte marker GFAP was performed by Real-time PCR. (C,D) CSC or U343MG adherent cells were seeded at different cell/well density, after 14 days the formed neurospheres were scored. (C) The graphs represent the logarithm of non-responding wells observed for the indicated cells/well density and (D) the total number of neurospheres formed for the indicated cells/well density (right panel). (E) U343MG cells or U343MG-CSCs were incubated with TMZ (250 µM) for 72 h. Then, cell proliferation was measured using the MTS assay. The data are presented as the mean values from three independent experiments, each performed in duplicate. ***P ≤ 0.001 vs U343MG. ## P ≤ 0.01 vs the relative effect in U343MG cells.

**Figure S3**

**Supplementary Figure 3**. Characterization of CSCs derived from T98G cells. (A) Representative bright field microscope images showing T98G -CSCs, bar correspond to 100 µm. (B) The total RNA was extracted from adherent T98G cells and from the derived CSCs. The relative mRNA quantification of the stem cell markers (CD133, Nanog, Nestin and OLIG2) and of the astrocyte marker GFAP was performed by Real-time PCR. (C,D) CSC or T98G adherent cells were seeded at different cell/well density, after 14 days the formed neurospheres were scored. (C) The graphs represent the logarithm of non-responding wells observed for the indicated cells/well density and (D) the total number of neurospheres formed for the indicated cells/well density (right panel). (E) T98G cells or T98G -CSCs were incubated with TMZ (250 µM) for 72 h. Then, cell proliferation was measured using the MTS assay. The data are presented as the mean values from three independent experiments, each performed in duplicate. ***P ≤ 0.001 vs U343MG. # P ≤ 0.05 vs the relative effect in T98G cells.

**Figure S4**

**Supplemental Figure 4.** Effects of CAR on U87MG and U343MG cell viability. Cells were treated in complete medium with the indicated concentrations of CAR for 7 days; the data were analysed with a non-linear regression. At the end of the treatments, cell proliferation was measured using MTS assay, as described in the Methods section. The data were expressed as a percentage with respect to untreated cells (control), which was set to 100%, and they are the mean values ± SEM of three independent experiments, each performed in triplicate.

**“Full-length blots relative to the cropped images showed in the main Figures”**

**Figure 1**


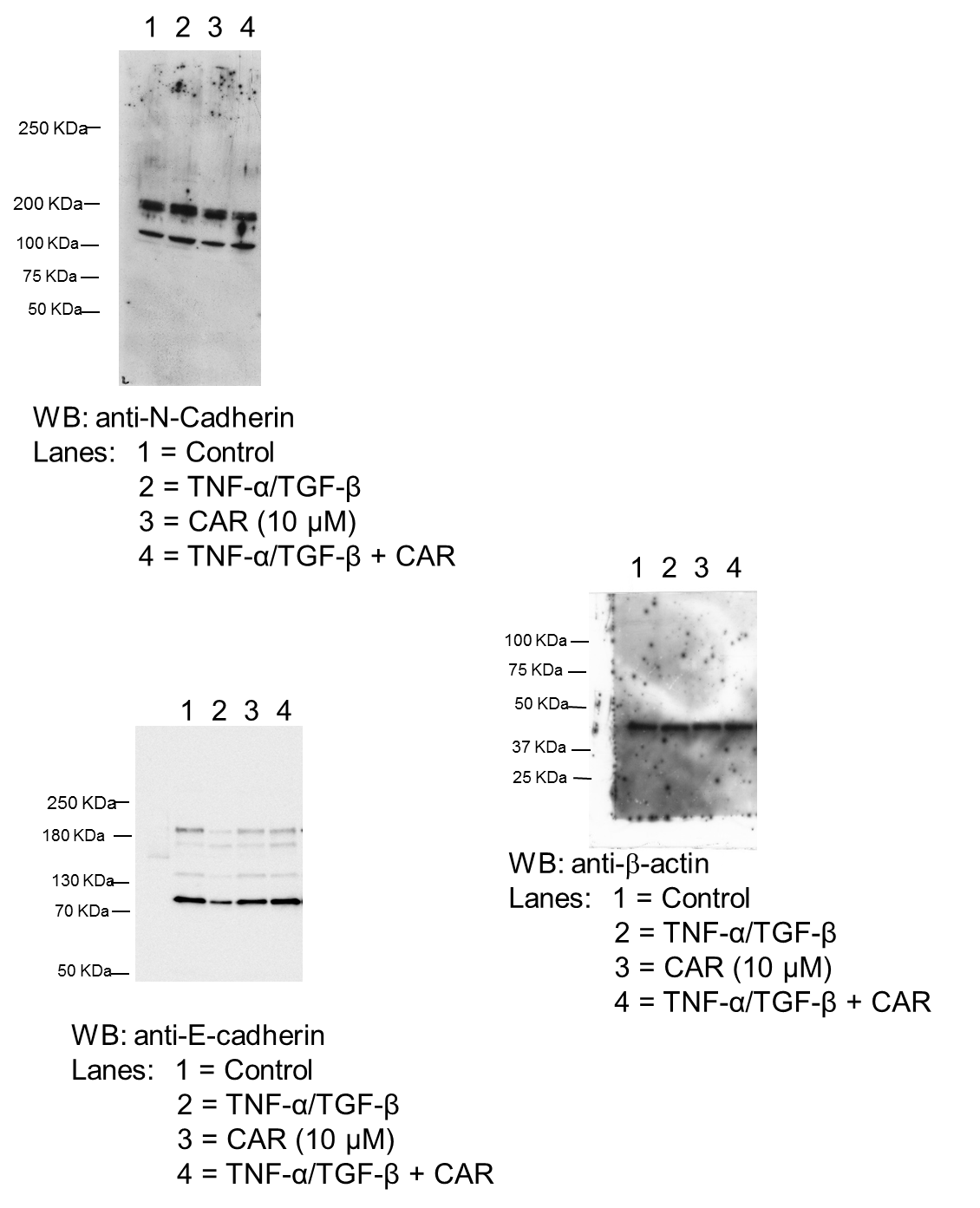


**
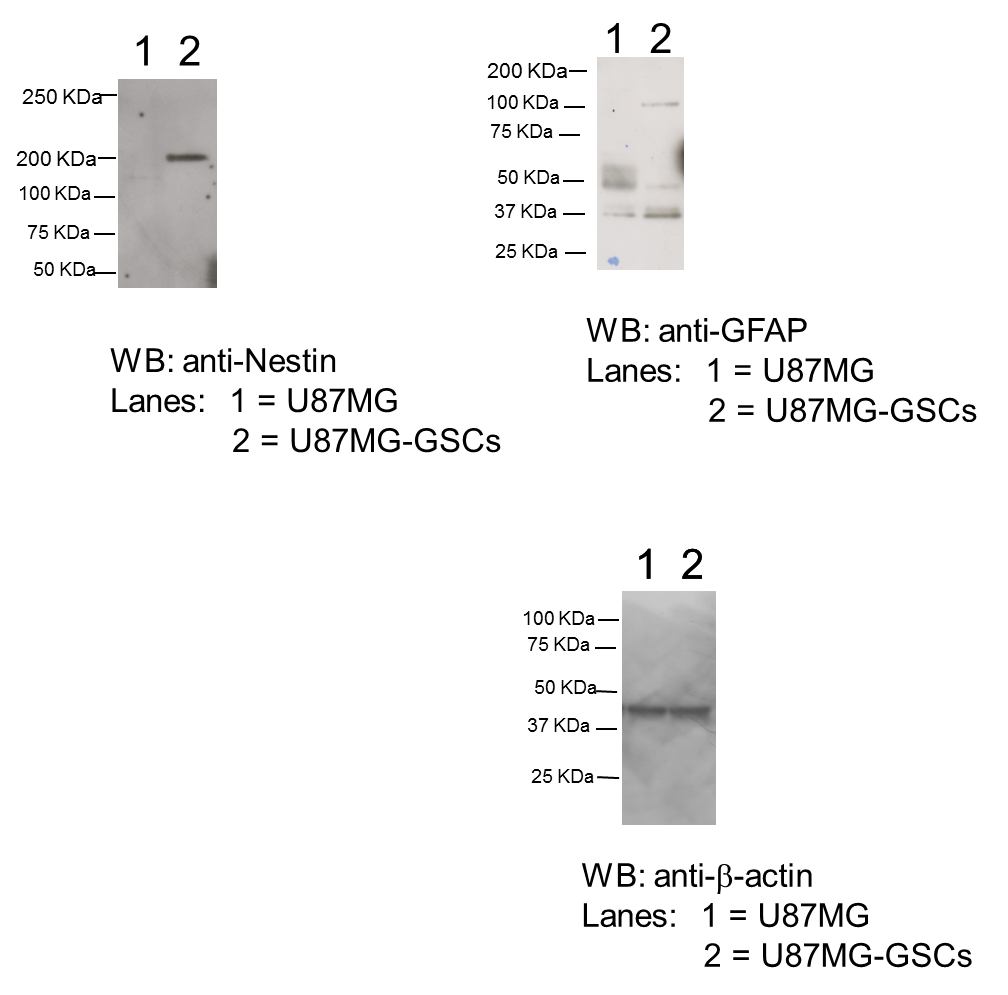
**
